# Clinical Evaluation of CyberKnife in the Treatment of Vestibular Schwannomas

**DOI:** 10.1155/2013/297093

**Published:** 2013-11-10

**Authors:** Jo-Ting Tsai, Jia-Wei Lin, Chien-Min Lin, Yuan-Hao Chen, Hsin-I Ma, Yee-Min Jen, Yi-Hsun Chen, Da-Tong Ju

**Affiliations:** ^1^Department of Radiation-Oncology, Taipei Medical University Wan-Fang Hospital, Taipei 110, Taiwan; ^2^Department of Radiation-Oncology, Taipei Medical University Shuang-Ho Hospital, New Taipei City 23561, Taiwan; ^3^Department of Neurosurgery, Taipei Medical University Wan-Fang Hospital, Taipei 110, Taiwan; ^4^Department of Neurosurgery, Taipei Medical University Shuang-Ho Hospital, New Taipei City 23561, Taiwan; ^5^Department of Neurological Surgery, Tri-Service General Hospital, National Defense Medical Center, No. 325, Section 2, Chenggong Road, Neihu District, Taipei 114, Taiwan; ^6^Department of Radiation Oncology, Tri-Service General Hospital, National Defense Medical Center, Taipei 114, Taiwan; ^7^Stereotactic Radiosurgery Center, Tri-Service General Hospital, National Defense Medical Center, Taipei 114, Taiwan

## Abstract

*Objective*. This study assessed the posttreatment tumor control and auditory function of vestibular schwannoma (VS) patients after CyberKnife (CK) and analyzed the possible prognostic factors of hearing loss. *Methods*. We retrospectively studied 117 VS patients, with Gardner-Robertson (GR) classification grades I to IV, who underwent CK between 2006 and 2012. Data including radiosurgery treatment parameters, pre- and postoperative tumor size, and auditory function were collected and examined. *Results*. With CK, 117 patients had excellent tumor control rates (99.1%), with a mean imaging followup of 61.1 months. Excluding 52 patients (GR III-IV pretreatment), 53 (81.5%) of the remaining 65 patients (initial GR I-II) maintained GR I or II hearing after CK, with a mean audiometric followup of 64.5 months. Twelve patients experienced hearing degradation (91.6% were GR II pretreatment); they appeared to have significantly larger tumor sizes, significantly smaller cochlear sizes, and higher prescribed cochlear doses, compared to the patients with preserved hearing. *Conclusion*. Our data showed that CK treatment provided an excellent tumor control rate and a comparable hearing preservation rate in VS patients. Patients with pretreatment GR II hearing levels, larger tumor volumes, smaller cochlear sizes, and higher prescribed cochlear doses may have poor hearing prognoses.

## 1. Introduction

Vestibular schwannoma (VS) accounts for 6–10% of all brain tumors and is a histopathologically benign tumor, commonly arising from the sheath of cranial nerve VIII [1]. The incidence of diagnosed VS has steadily increased in Denmark over time, from 3.1 tumors per million per year in 1976 to 19.4 per million per year in 2008 [2]. When the tumors grow, they compress cranial nerves VII, VIII, and V, as well as the brainstem, causing tinnitus, hearing loss, dizziness, vertigo, and gait instability [1, 3]. Today, viable treatment options for VS include observation, microsurgery, and radiation therapy, and the optimal indication for each individual should be determined on the basis of the size and location of the tumor, as well as the hearing level and patient age [1, 4, 5].

Over the past few decades, stereotactic radiosurgery (SRS) is commonly used to treat patients with small VS tumors, with the primary goal of tumor control. There are several adaptations of SRS, such as gamma knife and, more recently, linear accelerator radiosurgery (LINAC). SRS has been shown to be an effective alternative to microsurgery for small- and medium-sized VS, with tumor control rates of 93–100% [6–12]; however, its posttherapeutic hearing preservation rates range from 50 to 79% [7–13] and thereby remain unsatisfactory. In order to preserve posttreatment hearing and improve quality of life, some prognostic factors associated with hearing preservation, such as age at treatment, cochlear radiation dose, pretreatment hearing, tumor coverage, and so forth, are still under investigation [6, 8, 14, 15]. 

The CyberKnife (CK), which was first introduced by John Adler in 1994, is a dedicated robotic LINAC-based system, with the features of real-time image guidance, no rigid immobilization, and nonisocentric planning system [16]. In other words, the CK system is an improvement over the prior frame-based and single-staged techniques for patients with VS, but its clinical outcomes and risk factors in VS patients are still limited to the best of our knowledge [15, 17–19].

Thus, we performed this retrospective study to evaluate the tumor control, hearing outcomes, and possible prognostic factors of hearing loss in VS patients treated with CK, by using a fixed marginal dose of 1800 cGy in 3 sessions.

## 2. Materials and Methods

### 2.1. Subjects and Populations

This registry study was designed as a non-interventionist study in 2 medical centers in Taiwan, based on a therapeutic strategy and a retrospective patient chart review. The study was conducted in accordance with the Declaration of Helsinki-Good Clinical Practice. The protocol and study-related forms were both reviewed and approved by the Institutional Review Boards of the 2 medical centers in Taiwan.

This retrospective chart review was performed for patients who had undergone CK for unilateral VS, between 2006 and 2012, excluding those with neurofibromatosis type 2, those without hearing (Gardner-Robertson classification [GR] V) [20] prior to radiosurgical treatment, and patients without complete audiograms and magnetic resonance imaging (MRI) followups. Of the 117 cases, 24 had relapsed or had residuals after having prior treatments, and the remaining had primary SRS.

### 2.2. Radiosurgery Technique

Each patient was comfortably immobilized on the CyberKnife treatment table (Accuray, Sunnyvale, California) and wore a custom-made Aquaplast mask (WFR/Aquaplast Corp., Wyckoff, New Jersey). After an intravenous administration of 125 mL of Omnipaque contrast (350 mg I/mL; Nycomed, Inc., Princeton, New Jersey), a thin-slice high-resolution computed tomography (CT) and MRI T1 weighted image with contrast scan were obtained. Subsequently, the neurosurgeon outlined the tumor and its critical structures and generated a treatment plan with the CyberKnife Treatment Planning System (Multiplan v 2.1). Plans for CK treatment were evaluated using tumor coverage, homogeneity index (HI), conformity index (CI), and new conformity index (nCI). HI, CI, and nCI were calculated using the following formulas, respectively: HI =  *D*_max⁡_/prescribed dose, where *D*_max⁡_  is maximum dose; CI = prescription isodose volume (PIV)/tumor isodose volume (TIV), where PIV is total 3D volume of the isodose line and TIV is tumor volume covered by the isodose volume; nCI = tumor volume (TV) × plan target volume (PTV)/(target isodose volume)^2^.

### 2.3. Posttreatment and Followup

Baseline data, including patient characteristics (age, gender, tumor location, tumor volume, and cochlea size) and treatment of VS (date of radiosurgery and prescribed dose) were collected. Pre- and posttreatment hearing results were measured, according to pure tone averages (PTA) and speech discrimination scores, and classified using GR classifications [20]. Tumor size was measured by MRI in three orthogonal dimensions; therefore, tumor volume (Vol) was calculated using the following formula: Vol (mm^3^) = Tr(*a* × *b* × *c*)/6, where *a*, *b*, and *c* is width, height, and thickness, respectively [21]. When we compared the pretreatment MRI images to the last followup MRI images [17], if the tumor increased in size, we scored the patient as having tumor progression; otherwise, if the tumor either maintained its volume or decreased in volume, then we regarded this patient as having tumor control. Clinical evaluations and MRI images were performed at 3 months, 9 months, and 18 months after CK and then annually thereafter.

### 2.4. Statistical Analysis

Descriptive summaries were provided for the patients' demographics. The paired *t* test and Pearson Chi-square test were used to compare clinical parameters between groups, and a  *P*  value of <0.05 was considered statistically significant. 

## 3. Results

### 3.1. Patient Demographics

We reviewed the medical records of 117 patients (57 men and 60 women; average age, 57.3 years (range, 24–90 years)) with VS who had received CK between 2006 and 2012 (Table 1). Fifty-seven patients had right-sided tumors, while sixty patients had left-sided tumors. The mean tumor and cochlear sizes were 4739.2 mm^3^ (range, 23–19870 mm^3^) and 42.5 mm^3^ (range, 1–435 mm^3^), respectively. The mean duration of audiometric and imaging followups were 64.5 ± 17.3 months (range, 21–89 months) and 61.1 ± 16.8 months (range, 18–87 months), respectively.

The tumors were irradiated with a marginal dose of 1800 cGy in 3 sessions, which is equivalently equal to 1130 cGy (*α*/*β* = 3), with a 72–90% isodose line (mean 79.4%) and an average of 97.1% tumor coverage. Detailed cochlear dose-volume information is shown in Table 2. For example, 111 patients (94.9%) received 600 cGy (range: 600–630, mean: 608.6 ± 6.9 cGy) and their mean cochlear volume receiving 600 cGy was 83.6 ± 29.4 mm^3^.

### 3.2. Hearing Preservation

Of the 117 patients, 52 had nonserviceable or poor hearing (GR III-IV) pretreatment and were not counted in the following serviceable hearing preservation rates. As shown in Table 3, of the remaining 65 patients, all of whom had GR I to GR II hearing prior to treatment, after a mean audiometric followup of 64.5 months (range, 21–89 months), 53 (81.5%) patients had either maintained GR I or II hearing at the last follow-up visits, including 45 with unchanged hearing and 8 with worse but still serviceable hearing. The remaining 12 patients experienced hearing degradation, of which 11 (91.6%) had pretreatment GR II hearing. Comparing to the patients who had hearing retention, these 12 patients who had lower GR classifications (Table 4) had significantly larger tumor sizes of 6845.2 ± 6704.4 mm^3^ (range, 630–19870 mm^3^) (*P* < 0.001), significantly smaller cochlear sizes of 26.8 ± 13.8 mm^3^ (range, 8–59 mm^3^) (*P* < 0.001), and higher prescribed cochlear doses, with a maximum of 1525.7 ± 434.8 cGy (range, 1040–2591 cGy) and a minimum of 919.3 ± 484.1 cGy (range, 98–1866 cGy). Overall, larger tumor volumes and small cochlear volumes were significantly associated with hearing losses after SRS.

### 3.3. Tumor Control

After an average imaging followup of 61.1 months (range, 18–87 months), tumor reductions were detected in 20 patients (17.1%), while the tumors remained unchanged in 96 (82.0%) patients. There is one patient who had tumor progression (0.9%), and therefore an overall tumor control rate of 99.1% was achieved. 

## 4. Discussion and Conclusion

Surgical resection was once the traditional indication for VS. Today, however, continuous advancement in imaging technology and increased awareness of VS favor the early diagnosis of small- and medium-sized intracranial VS tumors. Therefore, the current management strategy for VS has shifted to observation, microsurgery, and radiation therapy, with an emphasis on the preservation of facial nerve function and hearing. Gamma radiosurgery, representing the gold standard in the SRS system, has been clinically proven to be effective in VS tumor control, but its hearing preservation rate, ranging from 55–79% [8, 22–26], is not satisfactory as a functional preservation-oriented treatment option. Recently, the CK system has emerged as a revolutionary treatment not only for VS but also for the whole body, owing to its robotic arm and computerized image processing, enabling real-time image guidance, and its dynamic tracking software, allowing for precise irradiation of the target volume. However, to our knowledge, published articles discussing the treatment of VS with CK are limited. Therefore, this study aimed to evaluate the clinical outcomes, including tumor control and hearing retention, and possible prognostic factors of hearing loss in VS patients treated with CK.

Tumor control has been achieved in VS patients treated with SRS, including those with the CK system. Tumor control rates had reached 100% in 15 months [27], 98% in 48 months [17], and 96% in 60 months [15] post-CK, revealing slightly decreasing, but still remarkable, tumor control rates over time, since the CK intervention. Consistent with these previous studies, our data also showed a 99.1% tumor control rate in 61 months, thereby confirming that CK had greatly contributed to tumor control in patients with VS. 

Aside from tumor control, hearing retention has been a substantial goal for CK treatment in VS. An early, small series reviewed 14 VS patients, initially with serviceable hearing (GR I or II) before CK, received mean marginal doses of 17 Gy in 1–3 sessions (prescribed dose 11.3 Gy). Thirteen of the fourteen cases retained serviceable hearing at the end of the followups, with functional hearing preservation rates of 93% [18]. More recently, 2 larger series respectively, analyzed 61 and 94 patients, both with serviceable hearing and mean, prescribed doses of 11.5–12 Gy, and reported the similar hearing preservation rates of 74%, within different mean followup periods (4 years versus 2.4 years) [15, 17]. We also demonstrated a serviceable hearing preservation rate of 81.5% at a mean followup of 64.5 months. Overall, CK provided comparable hearing preservation rates to the SRS system, ranging from 50–79% [7–13]. Nevertheless, the hearing preservation rate with SRS remains modest, and therefore numerous publications tried to verify the prognostic factors related to the hearing loss of VS in patients undergoing SRS. 

First of all, tumor characteristics are targets of interest to researchers for improving hearing prognosis. Several factors, such as tumor location, intracanalicular tumor volume, entire tumor volume or diameter, and tumor growth rate, were investigated recently. Our study found that the patients who developed hearing degradation were characterized as having significantly larger tumor volumes than those of patients with preserved hearing, which was in agreement with the study by Kano et al., [25] revealing that a tumor volume <0.75 cm^3^ was a significant prognostic factor for serviceable hearing preservation. However, several articles suggested that a tumor's volume or diameter does not appear to be a risk factor for hearing loss after SRS for VS [22, 23, 28, 29]. Yang et al. [29] systematically reviewed 45 publications (4,234 patients), reporting assessable and quantifiable outcome data in patients who underwent SRS for VS, and found that patients with small tumors (≤1.5 cm^3^) had similar hearing preservation rates (62%) as those with larger tumors (61%, *P* = 0.8968). As a whole, it remains difficult to draw a conclusion in terms of whether an increased tumor volume is a negative prognostic factor for functional hearing maintenance, owing to the diverse tumor volume thresholds chosen by researchers (0.75 cm^3^ versus 1.5 cm^3^) and the incomplete exclusion of possible influence from other tumor-related factors. 

In the present study, we showed that the patients who had diminished hearing after CK also had significantly smaller entire cochlear volumes those of patients with hearing preservation. The relationship between hearing preservation and cochlear volume has not yet been fully investigated. Massager et al. [26] reviewed 82 VS patients treated with gamma knife and reported that the median cochlear volumes were 80.9 mm^3^ in patients who had their hearing preserved, which was nonsignificantly higher than those of patients with hearing degradation (80.4 mm^3^). More recently, a study, containing 94 VS patients treated with CK, had demonstrated that cochlear volume was positively associated with hearing preservation [15], which resembles our results with CK. Overall, the independent role of cochlear volume, or aforementioned tumor volume, in the hearing preservation of VS patients after SRS remains unclear. It is recommended that more investigations be carried out for further verification. 

According to previous studies, a high radiation dose delivered to a cochlea is significantly associated with a worse hearing outcome [6, 8, 14, 15, 25, 26, 30, 31]. Similarly, we also demonstrated that both maximal and minimal radiation doses to the cochleas of patients with hearing deterioration were 1525.7 ± 434.8 cGy and 919.3 ± 484.1 cGy, respectively, which were higher than those of patients with hearing preservation (max: 1207.9 ± 409.3 cGy, min: 649.1 ± 304.9 cGy). It makes sense that doses prescribed to cochleas affect hearing after SRS because direct radiation may cause harm to the inner ear structures, especially the outer hair cells within the organ of Corti and the cells of the stria vascularis, resulting in further hearing damage [26]. Up to the present, the cochlear threshold dose has not yet been determined, but several investigators have provided some clues. Kano et al. [25] indicated that a patient receiving <4.2 Gy to the center of the cochlear had significantly better odds of maintaining the same hearing level. Massager et al. [26] showed that the median cochlear dose in patients with preserved hearing was 3.7 Gy, while that in patients with worsening of hearing was 5.33 Gy. Furthermore, Brown et al. [8] found that the mean percentage of the cochlear volume receiving ≥5.3 Gy was strongly associated with hearing loss. As Linskey [31] suggested, the cochlear dose probably lies somewhere between 4 Gy and 5.33 Gy, and we should carefully reduce the radiation dose to the cochlea, if possible. 

Observation is still a common treatment strategy for VS, especially for the elderly and those with small-sized tumors, owing to its noninvasive nature. However, there is increasing evidence for the benefits of early intervention. First, Yamakami et al. [32] systematically reviewed 903 patients with conservative management, over a 3.1-year period, and showed that one-third of these patients lost useful hearing. Régis et al. [24] further compared the hearing preservation results, between wait-and-see strategies and gamma knife treatments, illustrating that the useful hearing preservation rates at 3, 4, and 5 years were 75%, 52%, and 41%, respectively, in the wait-and-see group, and 77%, 70%, and 64%, respectively, in the gamma knife group, and concluded that the wait-and-see strategy raises the risk of hearing deterioration in patients with VS. 

Second, as our data showed, 91.6% of the 12 patients experiencing hearing degradation after CK had a pretreatment hearing status of GR II, reflecting a greater probability of hearing loss in patients with GR II hearing pretreatment. Similarly, several articles showed that patients with GR I hearing prior to SRS have better hearing prognoses posttreatment [6, 12, 23, 33]. Third, Yomo et al. [30] measured the annual hearing decrease rates (AHDR) before and after radiosurgery and found that the mean AHDR before SRS was 5.39 dB/year, compared with 3.77 dB/year after SRS, which revealed a reduction in the hearing loss rate after radiosurgery. Altogether, early intervention with SRS, especially when a patient still has good initial hearing, can lead to better hearing preservation. 

This study had few limitations. Given that hearing loss after radiotherapy is a late effect and that VS generally grows slowly, our audiometric and imaging followups may not have been long enough, which may have resulted in a better hearing outcome and tumor control rate. Additionally, to some extent, it could be difficult to accurately measure the cochlear dose because the location and size of VS could influence the tumor coverage generated by the treatment planning system. Diversified cochlear structures also biased the estimation of radiation doses to proximal critical organs, which may result in inaccurate calculations of the radiation doses. 

In treating VS, possible treatments have achieved similar tumor control outcomes, so the toxicity profiles should be primarily considered when deciding on treatments. In addition to fractionating a course of radiation treatment, improving the targeting, accuracy, and conformity of the prescribed dose are known to be helpful in mitigating radiation toxicities [4, 17]. The CK system not only functions as a staged radiosurgery that allows for a multisession dose regime but also has a higher accuracy compared with the LINAC-based system [4], suggesting that CK provides a favorable chance for preserving both hearing and neurocognitive functions. Therefore, CK is a promising treatment for VS and worthy of further investigation.

This study showed that VS patients receiving CK treatment achieved an excellent tumor control rate of 99.1%, with a comparable serviceable hearing preservation rate of 81.5%. Patients with posttreatment hearing deterioration were characterized as having GR II level hearing before CK, with larger tumor volumes and smaller cochlear sizes, and were prescribed higher doses to their cochleas. Overall, delivering 1800 cGy in 3 sessions by CK represents an effective and safe treatment option for the management of VS. 

## Figures and Tables

**Table 1:** Clinical characteristics and treatment of patients.

	Value
Patient characteristic	
Total no. of patients	117
Gender (M : F)	57 : 60
Mean age, y	57.3 (13.9)
Mean audiometric followup, mo	64.5 (17.3)
Mean imaging followup, mo	61.1 (16.8)
Tumor side, right/left	57/60
Previous surgery	24
Mean tumor size, mm^3^	4739.2 (5053.5)
Mean cochlear size, mm^3^	42.5 (46.1)
Cyberknife parameters	
Dose prescription isodose line, %	79.4 (3.7)
Coverage, %	97.1 (1.5)
CI	1.3 (0.1)
HI	1.3 (0.1)
nCI	1.3 (0.1)

Data are presented as mean (SD) or *N*.

**Table 2:** Cochlear dose-volume lists.

Radiation dose				Frequency distribution (%)	Cochlear volume (mm^3^)
cGy	Min.	Max.	Mean		
600	600	630	608.6 ± 6.9	111 (94.9%)	83.6 ± 29.4
800	800	824	810.5 ± 6.9	104 (88.9%)	65.0 ± 38.9
1000	1000	1025	1010.3 ± 7.6	92 (78.6%)	46.6 ± 41.6
1200	1200	1216	1208.2 ± 6.1	69 (58.9%)	30.9 ± 37.5
1400	1400	1421	1411.3 ± 7.2	51 (43.6%)	15.1 ± 27.9
1600	1600	1620	1612.1 ± 7.7	26 (22.2%)	6.2 ± 17.2
1800	1800	1822	1803.9 ± 7.3	7 (6.0%)	1.4 ± 9.8

**Table 3:** Hearing outcomes before and after treatment for patients with pretreatment GR I and II hearing.

Pretreatment	Posttreatment			
	GR I	GR II	GR III	Total
GR I	25	8	1	34
GR II	0	20	11	31

Total	25	28	12	65

**Table 4:** Comparison of patients with worse hearing and preserved hearing.

	Patients with worse hearing (*n* = 12)	Patients with preserved hearing (*n* = 53)
Tumor volume (mm^3^)	6845.2 ± 6704.4	4047.3 ± 4441.7*
Cochlear Volume (mm^3^)	26.8 ± 13.8	51.0 ± 64.9*
Dose to the cochlear (cGy)		
Maximum	1525.7 ± 434.8	1207.9 ± 409.3
Minimum	919.3 ± 484.1	649.1 ± 304.9

*P < 0.001 versus patients with worse hearing.
